# Synergistic effects of nitric oxide and exercise on revascularisation in the infarcted ventricle in a murine model of myocardial infarction

**DOI:** 10.17179/excli2015-510

**Published:** 2015-10-14

**Authors:** Kamal Ranjbar, Farzad Nazem, Afshin Nazari, Mohammadreza Gholami, Ali Reza Nezami, Malihe Ardakanizade, Maryam Sohrabi, Hasan Ahmadvand, Mohammad Mottaghi, Yaser Azizi

**Affiliations:** 1 Department of Sport Physiology, Faculty of Physical Education and Sport Sciences, Bu-Ali Sina University, Hamedan, Iran; 2 Department of Physiology, Razi Herbal Medicine Research Center, Lorestan University of Medical Sciences, Khorramabad, Iran; 3 Department of Anatomy, Lorestan University of Medical Sciences, Khorramabad, Iran; 4 Department of cardiology, Shahid madani hospital, Lorestan University of Medical Sciences, Khorramabad, Iran; 5 Department of Anatomy, School of Medicine, Hamadan University of Medical Sciences, Hamadan, Iran; 6 Department of Biochemistry, Faculty of Medicine, Lorestan University of Medical Sciences, Khorramabad, Iran; 7 Department of Physiology, Physiology research center, School of Medicine, Iran Universty of Medical Sciences, Tehran, Iran

**Keywords:** exercise training, L-arginine, myocardial infarction, angiogenesis, cardiac function

## Abstract

It has been shown that density of microvessels decreases in the left ventricular after myocardial infarction (MI). The change of angiogenic and angiostatic factors as the main factors in revascularisation after exercise training in area at risk is not determined yet in MI. Therefore, the aim of the present study was the effect of exercise training and L-arginine supplementation on area at risk angiogenesis in myocardial infarction rat. Four weeks after surgery (Left Anterior Descending Coronary artery Ligation), myocardial infarction rats were divided into 4 groups: Sedentary rats (Sed-MI); L-arginine supplementation (La-MI); Exercise training (Ex-MI) and Exercise + L-arginine (Ex+La). Exercise training (ET) lasted for 10 weeks at 17 m/min for 10-50 min day^−1^. Rats in the L-arginine-treated groups drank water containing 4 % L-arginine. After ET and L-arginine supplementation, ventricular function was evaluated and angiogenic and angiostatic indices were measured at ~1 mm from the edge of scar tissue (area at risk). Statistical analysis revealed that gene expression of VEGF as an angiogenic factor, angiostatin as an angiostatic factor and caspase-3 at area at risk decrease significantly in response to exercise training compared to the sedentary group. The capillary and arteriolar density in the Ex groups were significantly higher than those of the Sed groups. Compared to the Ex-MI group, the Ex+La group showed a markedly increase in capillary to fiber ratio. No significant differences were found in infarct size among the four groups, but cardiac function increased in response to exercise. Exercise training increases revascularization at area at risk by reduction of angiostatin. L-arginine supplementation causes additional effects on exercise-induced angiogenesis by preventing more reduction of VEGF gene expression in response to exercise. These improvements, in turn, increase left ventricular systolic function and decrease mortality in myocardial infarction rats.

## Introduction

Myocardial infarction (MI) is the most common cause of heart failure and high mortality rates and major impairment in quality of life. MI includes myocyte cell death due to loss of blood flow and ischemia. Cardiac remodeling after myocardial infarction involves cardiomyocyte hypertrophy, left ventricular (LV) systolic and diastolic dysfunction, apoptosis and reduction in nitric oxide bioavailability (Widder and Ertl, 2010[33]) and capillary density (de Waard et al., 2010[7]; Qin et al*.*, 2010[24]).

Reperfusion by revascularization is one of the most important steps in reducing ventricular remodeling after MI. Angiogenesis, the formation of new blood vessels from the preexisting vessels, is a complex process that plays an important role in revascularization and pathophysiology of post infarction ventricular remodeling and heart failure (Murohara & Asahara, 2002[20]). Angiogenesis is an alternative source of blood supply to myocardium jeopardized by ischemia and orchestrated by a balance between endogenous angiogenic and angiostatic factors (Athira et al*.*, 2013[1]). 

Vascular endothelial growth factor (VEGF) is a potent mitogen for vascular endothelial cells that regulates vessel formation by proliferation and migration of endothelial cells (Cebe-Suarez et al., 2006[4]; Forsythe et al., 1996[9]). VEGF expression is transcriptionally regulated by hypoxia which occurs during ischemia and myocardial infarction (Liu et al., 1995[17]; Forsythe et al., 1996[9]). On the other hand, angiogenesis can be inhibited at any of a number of key steps in the tube formation by angiostatic factors, such as angiostatin (Ruhrberg, 2001[27]; Nyberg et al., 2005[21]). Angiostatin is a cryptic fragment of plasminogen that inhibits endothelial cell proliferation and migration (Nyberg et al*.*, 2005[21]).

The molecular mechanism of angiogenesis in myocardial infarction is not yet clear. It is likely that different mediators are involved in different stages of angiogenesis. It was recently shown that nitric oxide (NO), which is synthesized from L-arginine via endothelial nitric oxide synthase (NOS), plays an important role in the regulation of angiogenesis. The release of endothelium-derived NO is reduced in ischemic heart disease patients (Murohara and Asahara, 2002[20]). It is then conceivable that NO-mediated angiogenesis is impaired in chronic heart failure. In fact, angiogenesis is attenuated when NO bioactivity is reduced (Cooke and Losordo, 2002[6]). The role of NO in angiogenesis is not fully elucidated. NO is an endothelial survival factor, inhibiting apoptosis by decreasing caspase-3, and enhancing endothelial cell migration and proliferation, perhaps in part by increasing the expression of VEGF (Cooke and Losordo, 2002[6]). Also, NO suppress the production of angiostatin (Matsunaga et al*.*, 2002[19]). On the other hand, exercise training attenuates LV dysfunction after myocardial infarction (Bansal et al., 2010[2]). The mechanisms to explain this benefit have not been fully delineated. 

Also, previous studies showed that apoptosis (programmed cell death) plays a pivotal role in the tissue damage after MI (Krijnen et al*.*, 2002[14]). In general, caspases form a key step in the process of apoptosis. Recent findings showed that Caspase-3 increased infarct size and a pronounced susceptibility to die (Condorelli et al., 2001[5]).

In this paper we tested this hypothesis that angiogenesis plays an important role in the improvement of LV function after exercise training in MI rats. Accordingly, this study was designed to evaluate the effect of aerobic exercise training and L-arginine supplementation on area at risk revascularization, caspase-3 and mortality rate in myocardial infarction rats.

## Methods

### Animals

Six to eight week-old male wistar rats (180 ± 210 g) were housed under standard conditions (22 ± 2 °C and 60 % humidity). They were provided with standard laboratory rat chow and had free access to water. These investigations were carried out in accordance with National Institutes of Health Guide for the Care and Use of Laboratory Animals, and the study protocols were accepted by animal's ethics committee at Lorestan University of medical science.

### Rat model of myocardial infarction

Myocardial infarction was created by permanent ligation of the left anterior descending coronary artery as described previously (Sun et al*.*, 2001[28]). Briefly, after intubation, left thoracotomy and pericardiotomy, 6-0 silk suture was placed around the left anterior descending coronary artery localized in 2 mm below the left atrium. The chest was closed and lung reinflated using positive end expiratory pressure. A computerized data acquisition system (ML750 Power Lab/4sp, AD Instruments) was used for monitoring ECG. ST-segment elevation and Q wave inversion were indicators of successful operation. Respiratory functions were preserved through use of a ventilator (Small Animal Ventilator, Model 683, Harvard Apparatus, 15 ml/kg stroke volume and 60-70 breaths/ min) and body temperature was maintained with an incubator that was fixed to a laboratory bench (Ranjbar et al*.*, 2015[25]).

### Experimental groups

Surviving rats, 4 week after surgery, randomly assigned to the following experimental groups: MI-sedentary (n=10, Sed-MI); MI-exercise (n=10, Ex-MI); MI-sedentary+L-arginine (n=10, La-MI); MI-exercise+L-arginine (n=10, Ex+La). The rats assigned to the exercise group started exercising at 4 weeks post-MI using a motorized rodent treadmill for 10 weeks, while the sedentary rats remained sedentary throughout the experiment period. During the study, drinking water without L-arginine was available in Sed-MI and Ex-MI groups.

### Drug treatment and exercise training

L-arginine treatment was initiated 4 weeks post-MI. Rats in the L-arginine-treated groups consumed 4 % L-arginine solution (w/v) (A5006, Sigma-Aldrich, USA) (Suzuki, 2005[29]). The determination of L-arginine dosage was based on the previous studies, which demonstrated positive effect in improving cardiac angiogenesis without symptoms of side effect (Suzuki, 2005[29]).

Rats exercised at 10 m/min, 5° incline for 10 min per session in the first week of training. Exercise intensity was gradually increased to 17 m/min and 50 min per session and maintained constant throughout the experiment. The exercise intensity was moderate and 55-60 % of maximal oxygen consumption (Bansal et al*.*, 2010[2]). This protocol was performed for 5 days a week (except Tuesdays and Fridays) for 10 weeks. The determination of treadmill speed and exercise duration was based on the previous studies (Xu et al*.*, 2008[34]; Bansal et al*.*, 2010[2]).

### Doppler echocardiography

Doppler echocardiographic mensuration was accomplished before (4 weeks post-MI) and after exercise training (14 weeks post-MI). Echocardiographic evaluations were performed by a blinded observer, under the guidelines of the American Society of Echocardiography. The rats were first anesthetized and sodium thiopental (50 mg/kg body weight, i.p.), their thoraces were shaved and the animals were positioned in the right lateral decubitus position. A Sonos 5500 equipment (Philips Medical Systems, Andover, MA, USA) with a 12-MHz transducer was used at a depth between 2 and 3 cm. The images were recorded on VHS videotapes and the final result was obtained from the mean of three different cardiac cycles. We measured the LV fractional shortening, LV ejection fraction, LV Stroke Volume and Cardiac Output. 

### Blood collection, tissue processing 

At the end of the echocardiography, (14 weeks after the operation) 48 h after the last exercise-training session, the rats were weighed, anesthetized and sacrificed with chloroform in a desiccator. After anesthesia, for measurement of serum NO, blood was collected via cardiac puncture. Blood samples were immediately centrifuged at 4 °C for 10 minutes at 1500 g and stored at -80 °C until analysis. After blood collection, heart was quickly excised, rinsed with ice-cold saline, blotted and weighed. After the atria and great blood vessels were trimmed, area at risk or border zone (~1 mm from the edge of scar tissue) from the antero-lateral free wall of the left ventricle was removed. Previous work has shown that sampling from this region is representative of the whole ventricle (Guth et al*.*, 1987). Tissue samples quickly were frozen in liquid N2 for gene analyses. Duration of the process was less than 2 min. As well as for immunohistochemistry analyses, left ventricular tissues, after tissue processor, were embedded in paraffin. 

### Determination of NO concentration in serum

Serum NO concentration was measured according to Griess's method. Briefly, 50 μl of serum were added to Griess's reaction (1 % sulfanilamide 1 gr + 1 % N-1-naphthylethylenediamine Dihydrochloride 1 gr + acid phosphoric 2.94 ml) for 10 minutes at room temperature. Nitrite concentrations were determined by spectrophotometric analysis at 540 nm and compared with sodium nitrite standards. The lower limit of sensitivity of this assay is 0.5 nmol/mL. NO products were expressed as *μ*mol/ml (Ratajczak-Wrona et al*.*, 2013[26]).

### RNA, cDNA synthesis and real-time PCR

50 mg of frozen heart tissue were homogenized in TRI reagent (Sigma, St. Louis, MO) buffer, and RNA isolation was completed using total RNA purification kits (Jana Biosciense GmbH, Germany) following manufacturer's instructions. Briefly, tissue sample was ground, and the resulting powder was resuspended in 200 μL of TRIzol reagent. The suspension was then homogenized and incubated for 5 min at room temperature. The homogenate was extracted with 500 μL of chloroform, and after centrifugation (10000 g, 10 min, 4 °C) the aqueous phase was mixed with 300 μL of isopropanol. The resulting pellet was washed with 700 μL of ethanol and resuspended in 40-50 μL of RNase-free water. Total RNA samples were stored at −80 °C until use.

Total RNA (1 μg) was reverse transcribed to complementary DNA using cDNA synthesis kit (AccuPower^®^ RT PreMIX, BIONEER, USA) according to the manufacturer's instructions.

Quantitative real-time PCR was conducted using SYBR Green PCR Master Mix Kit (Applied Biosystems, USA) to measure the expression of VEGF, angiostatin and caspase-3. Real-time PCR was measured by Rotor Gene 3000 following program: step 1: 95 °C for 20 sec, 3 min and step 2: 40 cycle of 95 °C for 5 sec and 60 °C for ˃ 20 sec. The mRNA expression was assessed by oligonucleotides primers for analysis of the genes VEGF-A (F:5ʹ- ATC TTT CAT CGG ACC AGT CG-3ʹ; R: 5ʹ- CCC AGA AGT TGG ACG AAA AG-3ʹ), angiostatin (F: 5ʹ- GAC CTC TGG TTT GCT TCG AG-3ʹ; R: 5ʹ-TTG GTT TGA TTG CTG TCA GG-3ʹ), caspase-3 (F: 5ʹ-CAG AAG CTC CTG CAA AAA GG-3ʹ; R: 5ʹ-AGT CTG CAG CTC CTC CAC AT-3ʹ) and β-actin (F: 5ʹ-AGC CAT GTA CGT AGC CAT CC-3ʹ; R: 5ʹ- CTC TCA GCT GTG GTGGTG AA-3ʹ). The relative expression of VEGF, angiostatin and caspase-3 mRNA was normalized to the control β-actin using the comparative threshold cycle (2^−ΔΔCt^) method. Each sample was analyzed in triplicate. Results are expressed in fold changes of control group.

### Immunohistochemistry and histological analyses

Transverse 5 μm-thick serial sections were cut from paraffin-embedded LV slices and mounted onto microscope slides. Hematoxylin and eosin (H & E) stain was used for capillary density evaluation. The capillary density was determined by counting the total number of capillary was expresses as the number of capillaries per square millimeter (Wagatsuma et al*.*, 2005[31]). 

Additional sections were either immunostained with α-actin smooth muscle primary antibodies (SantaCruz Biotechnology, Santa Cruz, CA, USA) to visualize the arterioles. All sections were counterstained with hematoxylin to visualize the cell nuclei. The stained sections were examined under the Olympus BX53 microscope (Shinjuku, Tokyo, Japan). SM α-actin-positive vessels were used to calculate the arteriolar number density. Arterioles were defined as vessels with an internal diameter in the range of 10-150 μm that had at least one layer of smooth muscle cells. All parameters were estimated separately for the area at risk region. To determine capillary density, capillary to fiber ratio and arteriole density, the number of capillary, arteriole and myocyte was counted in a blind fashion in 10 fields persection of the border zone or area at risk at x 200 magnification and normalized to the section area.

### Infarct size measurement

Prior to sacrifice, 1 ml of Evans blue was injected through the femoral artery. Hearts were removed immediately and held for 24 hours at -70 °C. afterwards, the hearts sliced into 1 mm cross sections and incubated at 37 °C for 20 min using 1 % 2,3,5-triphenyltetrazolium chloride in 0.1 M phosphate buffer. Then the heart slices were fixed in 10 % formaldehyde for 24 hours and samples scanned by scanner (HP Scanjet G2410 Flatbed Scanner). Infarct size was measured by using Photoshop software (Briet et al*.*, 2008[3]).

### Statistical analysis

Statistical calculations were performed using SPSS version 20.0. Descriptive data (means ± SEM) were calculated for each dependent variable. The normal distribution of all data was approved by Shapiro-wilk test. Overall group differences were analyzed using a one way ANOVA. When appropriate, post-hoc analyses were made using a Tukeys HSD test. To evaluate of the % of survival after MI, Kaplan-Meier survival analysis was used. The significance level was set at p ˂ 0.05.

## Results

### General characteristics

Table 1(Tab. 1) presents the characteristics of animals included in the studies at the end of protocol. Heart rate (at rest), body weight, heart weight and heart weight to body weight ratio were not significantly different between groups.

### Nitric oxide

**Figure 1:**
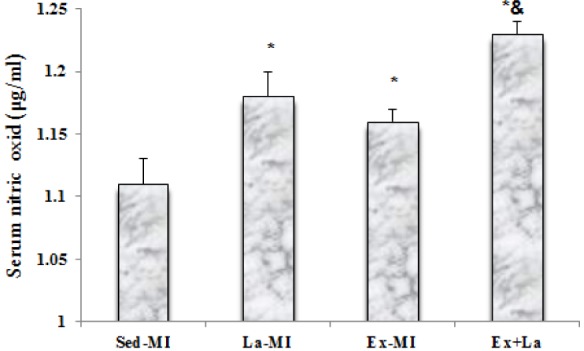
Level of serum nitric oxide in different groups. * Significant difference from the Sed-MI group at the level of P < 0.05, & significant difference from the Ex-MI group at the level of P < 0.05.

As shown in Figure 1(Fig. 1), the serum NO levels in both L-arginine treated groups (La-MI, Ex+La) and Ex-MI group were significantly increased compared to the Sed-MI group. The serum NO level was significantly higher in the Ex+La group than in the Ex-MI group, but there was not a significant difference between the Ex-MI and La-MI groups.

### Echocardiographic data

10 week aerobic exercises training with and without L-arginine supplementation improved left ventricular systolic function in myocardial infarction rat and prevented cardiac function deterioration after MI. LV ejection fraction (LVEF) was increased in exercise training groups in comparison to the sedentary groups. However, LVEF was similar in Ex-MI and Ex+La groups. On the other hand, exercise training significantly increased fractional shortening (FS), stroke volume (SV) and cardiac output (CO) compared to the sedentary infarcted groups (Sed-MI, La-MI). FS, SV and CO was significantly higher in the Ex+La group than Ex-MI group (Table 2(Tab. 2)). 

### Gene expressions in the border zone left ventricular

**Figure 2:**
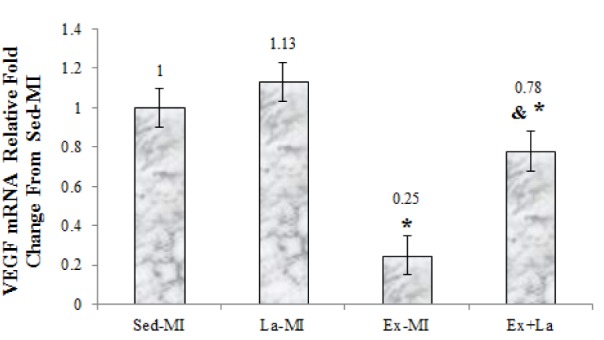
The graph represents the relative expression levels of VEGF mRNA, after exercise training and L-arginine treatment, compared to Sed-MI animals. * Significant difference from the Sed-MI group at the level of P < 0.001. & Significant difference from the Ex-MI group at the level of P < 0.05

**Figure 3:**
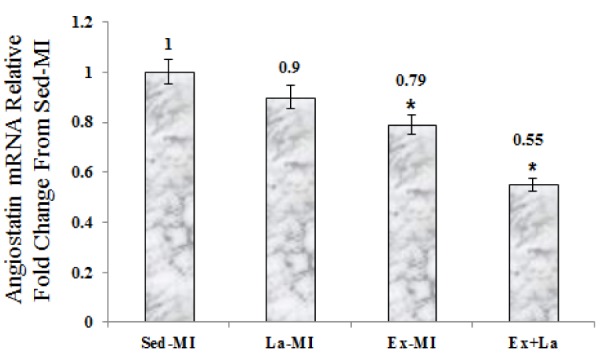
The graph represents the relative expression levels of angiostatin mRNA, after exercise training and L-arginine treatment, compared to Sed-MI animals. * Significant difference from the Sed-MI group at the level of P < 0.01

**Figure 4:**
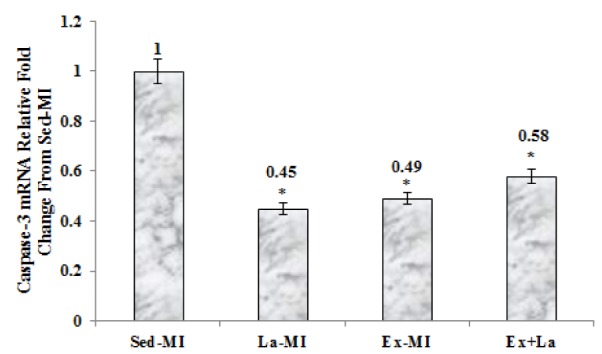
The graph represents the relative expression levels of caspase-3 mRNA, after exercise training and L-arginine treatment, compared to Sed-MI animals. * Significant difference from the Sed-MI group at the level of P < 0.001

As seen in Figures 2-4(Fig. 2)(Fig. 3)(Fig. 4), we found a significant difference in mRNA level of VEGF, angiostatin and caspase-3 among the infarcted groups. Statistical analyses showed that a 10 week aerobic exercise training with or without L-arginine supplementation decreased VEGF, angiostatin and caspase-3 gene expression in area at risk noticeably. L-arginine supplementation did not significantly change gene expression of VEGF and angiostatin but it significantly decreased gene expression of caspase-3 compared to the Sed-MI group. The mRNA levels of VEGF were significantly higher in the Ex+La group than in the Ex-MI group. Furthermore, angiostatin and caspase-3 mRNA did not reveal a significant difference between the Ex-MI group and the Ex+La group.

### Immunohistochemistry

**Figure 5:**
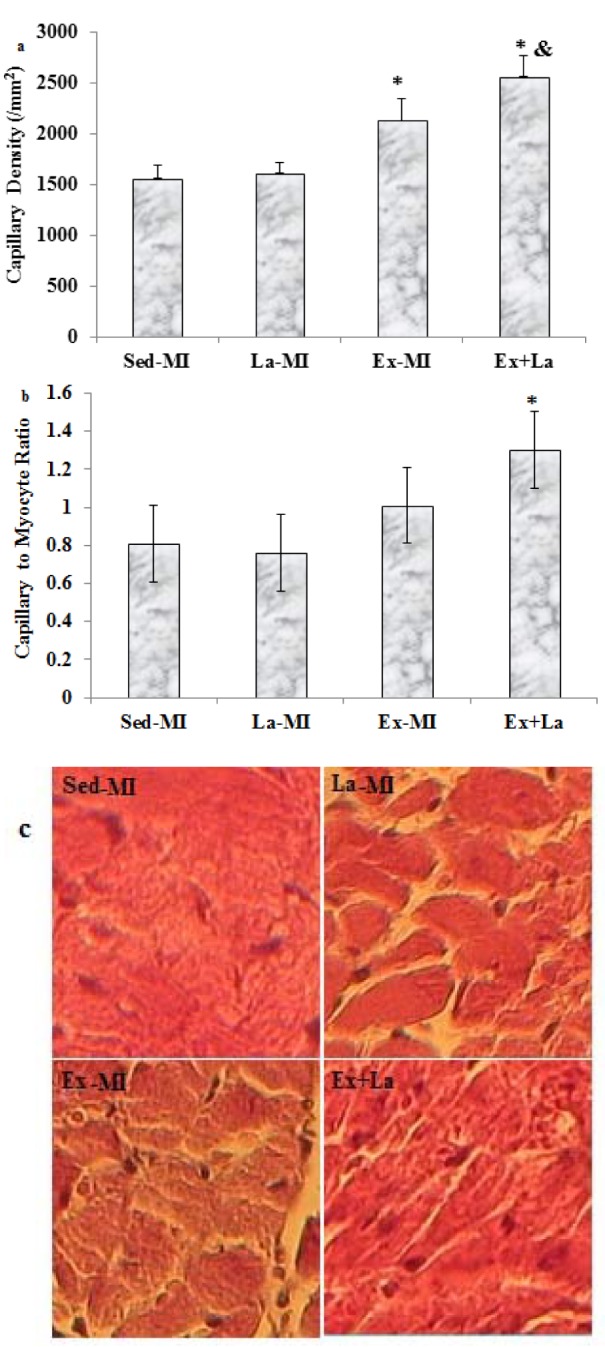
(a) Effects of exercise on area at risk capillary density. (b) The capillary to fiber ratio at area at risk. (c) Microscopic representative images of area at risk capillary diensity stained with H & E. Scale bar represents 100 µm, original magnification x 200. * Significant difference from the Sed-MI group at the level of P < 0.05, & significant difference from the Ex-MI group at the level of P < 0.05

Capillary density of area at risk significantly increased in response to exercise training. Training with L-arginine supplementation led to a further increase in comparison to the Ex-MI group. However, no significant difference was found between the Sed-MI and La-MI groups in the capillary density (Figure 5(Fig. 5)). 

**Figure 6:**
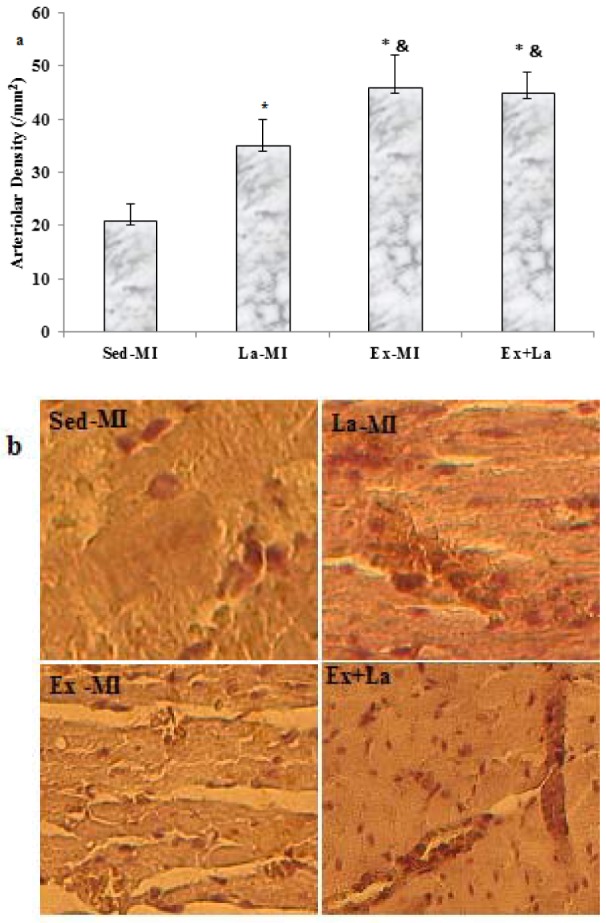
(a) Effects of exercise on area at risk arteriolar density. (b) Representative images of arterioles stained with antibodies against smooth muscle (SM) α-actin obtained from heart failure sedentary and exercised rats at 14 weeks after MI in the antero-lateral free wall of the left ventricle. Scale bar represents 100 µm, original magnification x 200. * Significant difference from the Sed-MI group at the level of P < 0.05, & significant difference from the Ex-MI group at the level of P < 0.05

Training with L-arginine supplementation markedly increased the capillary-to-ﬁbre (C/F) ratio in the area at risk, whereas training by itself did not (P = 0.07) (Figure 5(Fig. 5)). Furthermore, the arteriolar density in the Ex+La group was similar to that of the Ex-MI group, but it was significantly higher than that of the Sed groups (P<0.05). Also, L-arginine supplementation alone increased arteriolar density compared to the Sed-MI group (Figure 6(Fig. 6)).

### Infarct size 

**Figure 7:**
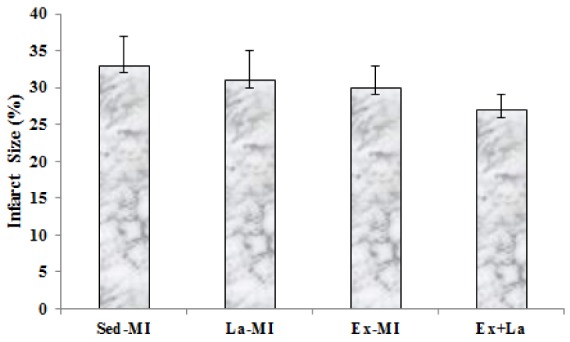
No significant differences were found in infarct size among the four groups.

Infarct size at the end of treatment was not signiﬁcantly different among the four groups. Infarct size was reduced in response to exercise, but these changes were not significant (Figure 7(Fig. 7)).

### Mortality

**Figure 8:**
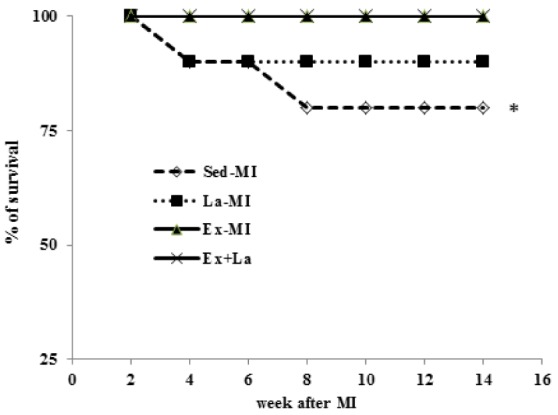
Survival rate at 14 weeks following myocardial infarction. Kaplan-Meier analysis revealed a trend of lower mortality in the training groups compared with the Sed groups. * Significant difference from the Ex groups at the level of P < 0.05

Figure 8(Fig. 8) shows the % of survival rate (Kaplan-Meier's survival curve) all of the subjects in this study. During the experimental protocol, mortality rate was higher in the Sed groups compared with the training groups (no deaths).

## Discussion

Growing evidence indicates that exercise can attenuate the LV deterioration and improve cardiac function after MI. But the molecular mechanisms of improved left ventricular function after MI in response to exercise training are not completely understood. MI is associated with a revascularisation response, which is crucial for healing and cardiac repair. 

In this study, we hypothesized that exercise training and L-arginine supplementation might favorably affect the performance of the failing heart through amelioration of revascularisation at area at risk and restoration of cardiac function.

The results of the study displayed 

(i) an increase of revascularisation in area at risk by decreasing angiostatin, 

(ii) that L-arginine has an additional effect on exercise-induced angiogenesis by preventing more reduction of VEGF gene expression in response to exercise training, 

(iii) a slight reduction in infarct size by reducing caspase-3 and 

(iv) an improvement of LV systolic function. 

These beneﬁts resulted in a reduction of mortality rate in trained infarcted animals.

VEGF is a powerful activator of endothelial proliferation and is crucial for nearly all forms of neovascularization (Gielen et al., 2010[12]). The results showed that the VEGF mRNA decreases in response to aerobic exercise training in area at risk. This result was not in agreement with the ﬁndings of Jorge et al. (2010[13]) who reported increase VEGF mRNA following 12 weeks exercise training in myocardial infarction rat. A probable explanation for the difference between the results of this study and the ﬁndings of Jorge et al. (2010[13]) related to the VEGF measurement location. 

The mechanism behind VEGF gene expression reduction at area at risk in response to exercise is unclear and complicated. Akt1 signaling controls VEGF synthesis. Early after MI, VEGF and Akt were strongly activated simultaneously, but in the chronic phase, Akt activation was lasted, while VEGF activation was reduced. Leosco and coworker showed the switch-off of Akt-VEGF pathway after 10 weeks of training in myocardial infarction rat. These change probably due to the improved myocardial vascularization and reperfusion of HF heart (Leosco et al., 2007[16]). Increase capillary density and C/F ratio, by increasing capillary exchange area, contributes to increased blood flow and increased oxygen uptake (O_2_ transport, conductance and extraction), decrease hypoxic tension in the local area, which reduced the expression of VEGF by reflex. 

For the first time in this study we showed that angiostatin significantly decreased in response to exercise training after MI. Reduction of angiostatin in response to exercise, is not yet clear. Because of this inverse relationship between MMPs activity and NO production (Matsunaga et al., 2002[19]), probable reductions of MMPs reduce release of angiostatin from plasminogen after exercise training and L-arginine supplementation. Matsunaga et al. (2000[18], 2002[19]) showed that cardiac collateralization was dependent on NO, which in part would severely restrict the activity of MMPs and the subsequent production of angiostatin from plasminogen. In this study although NO in Ex+La group is higher than Ex group, but angiostatin was not significant different between exercise training groups. Probably a difference of 0.06 µg/ml NO between two groups, does not affect angiostatin expression. 

Capillary density and C/F ratio were promoted in area at risk after exercise training and L-arginine supplementation. Training with L-arginine increased signiﬁcantly the C/F ratio, whereas training alone did not. The results from the present study suggest that L-arginine supplementation prevented more reduction VEGF gene expression in response to exercise. This results was in line with the ﬁndings of Leosco et al. (2007[16]) and Suzuki (2005[29]) that reported increase angiogenesis and arteriogenesin response to exercise with (Suzuki, 2005[29]) and without (Leosco et al*.*, 2007[16]) L-arginine supplementation. 

While the capillary network is important for oxygen delivery to cardiac myocytes, the coronary arteriolar bed is critical for a distribution of blood between capillary domains (Dedkov et al*.*, 2014[8]). We found higher arteriolar density in the Ex and Ex+La hearts and no difference between the Ex and Ex+La groups, suggesting that exercise training enhanced arteriolar density in area at risk after MI.

The mechanisms involved in the growth and remodeling of arterioles are generally unknown. In fact the processes of new capillaries formation are different from processes of new arterioles formation. It is clear that the factors promoting angiogenesis are different with those inducing capillary arterialization (Laughlin and Roseguini, 2008). New arteriole appearance when mature capillaries become surrounded by smooth muscle cell (Price et al*.*, 1994[22]; Price and Skalak, 1996[23]). In this regard White and coworkers (White et al., 1998[32]) suggested that early increase capillary density (observed at 3 weeks) after exercise training were the source of the new arterioles. Additional work will be required to ascertain the role of angiogenic and angiostatic factors and “capillary arterialization” in vascular adaptation induced by exercise training and NO in area at risk after MI.

The present study provides evidence that exercise training, despite the significant reduction of caspase-3 and increase microvessels density, is incapable of influencing infarct size in myocardial infarction rat. Furthermore, scattered fibrosis, apart from the infarct region, that detected after stressful exercise were not found (Gaudron et al*.*, 1994[11]). A previous study showed that infarct size reduction in response to exercise training seems to occur by opioid receptors and not by revascularisation (Galvão et al*.*, 2011[10]). It should be noted that angiogenesis and arteriolargenesis could supply more oxygen and nutrients to the cardiomyocytes in the border zones of MI, which could partly aid in the recovery of cardiac function after MI by ameliorating cardiomyocytes function (Tang et al., 2011[30]).

The molecular mechanisms of morbidity and mortality reduction after MI in response to exercise training have not been investigated. Although blood flow was not measured in our experiments, it is likely that increase in arteriolar density and capillary density permitted a morphometric basis for increased cardiac blood flow capacity exercise hearts that could help preserve the surviving myocardium in these hearts.

## Conclusion

In summary, in this study we showed that aerobic exercise training and L-arginine supplementation increase microvessles density at area at risk after MI by angiostatin reduction. L-arginine has an additional effect on exercise-induced angiogenesis by preventing more reduction of VEGF gene expression in response to exercise training. These improvements, in turn, increase left ventricular systolic function and decrease mortality in myocardial infarction rats.

## Conflict of interest

The authors declare that there are no conflicts of interests.

## Figures and Tables

**Table 1:**
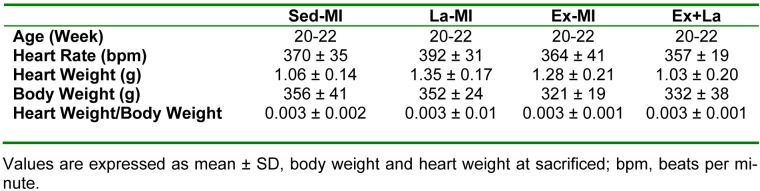
General characteristics at the end of protocol

**Table 2:**
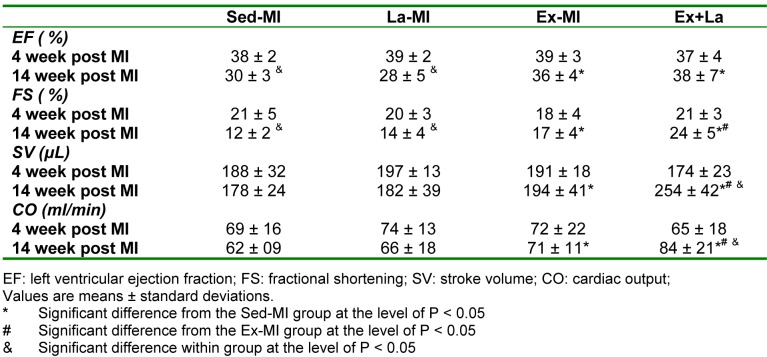
Doppler echocardiographic assessment of left ventricular geometry and function at 4 and 14 weeks post MI
